# Impact of Primary Staging with Fibroblast Activation Protein Specific Enzyme Inhibitor (FAPI)-PET/CT on Radio-Oncologic Treatment Planning of Patients with Esophageal Cancer

**DOI:** 10.1007/s11307-020-01548-y

**Published:** 2020-10-15

**Authors:** J. Ristau, F. L. Giesel, M. F. Haefner, F. Staudinger, T. Lindner, A. Merkel, J. Schlittenhardt, C. Kratochwil, P. L. Choyke, K. Herfarth, J. Debus, U. Haberkorn, S. A. Koerber

**Affiliations:** 1 Department of Radiation Oncology, Heidelberg University Hospital, Im Neuenheimer Feld 400, 69120 Heidelberg, Germany; 2 Heidelberg Institute of Radiation Oncology (HIRO), Heidelberg University Hospital, Heidelberg, Germany; 3 National Center for Tumor diseases (NCT), Heidelberg University Hospital, Heidelberg, Germany; 4 German Cancer Consortium (DKTK), Core Center Heidelberg, Heidelberg University Hospital, Heidelberg, Germany; 5 Department of Nuclear Medicine, Heidelberg University Hospital, Heidelberg, Germany; 6 Clinical Cooperation Unit Nuclear Medicine, German Cancer Research Center (DKFZ), Heidelberg, Germany; 7 Molecular Imaging Program, Center for Cancer Research, National Cancer Institute, National Institutes of Health, Bethesda, MD USA; 8 Department of Radiation Oncology, Heidelberg Ion-Beam Therapy Center (HIT), Heidelberg University Hospital, Heidelberg, Germany; 9 Clinical Cooperation Unit Radiation Oncology, German Cancer Research Center (DKFZ), Heidelberg, Germany; 10 Translational Lung Research Center Heidelberg (TLRC), German Center for Lung Research (DZL), Heidelberg, Germany

**Keywords:** FAPI, PET, Esophageal cancer, Fibroblast activation protein, Oncological management

## Abstract

**Purpose:**

Quinoline-based ligands targeting cancer-associated fibroblasts have emerged as promising radiopharmaceuticals in different tumor entities. The aim of this retrospective study was to explore the potential of FAPI-PET/CT in the initial staging of esophageal cancer patients and its usefulness in radiotherapy planning as a first clinical analysis.

**Methods:**

Seven patients with treatment-naive esophageal cancer underwent FAPI-PET/CT. Tracer uptake was quantified by standardized uptake values (SUV)max and (SUV)mean. Six patients received definitive and one neoadjuvant (chemo)radiation therapy. Endo-esophageal clipping, the gold standard to define tumor margins not delineable per CT, was performed in three patients.

**Results:**

Primary tumors demonstrated high FAPI uptake with a median SUVmax of 17.2. Excellent tumor-to-background ratios resulted in accurate target volume delineation and were found in perfect match with clipping. Detection of regional lymph node metastases facilitated the use of simultaneous integrated boost radiotherapy plans for these patients.

**Conclusion:**

FAPI-PET/CT may be beneficial for the management of esophageal cancer particularly in planning radiotherapy, but further research is necessary to increase patient number and statistical reliability.

## Introduction

Contrast-enhanced CT imaging of the chest and abdomen is part of the standard assessment of patients with newly diagnosed esophageal cancer. Diagnostic accuracy of T staging with CT, however, is limited as measurements of wall thickness can be difficult. FDG-PET/CT imaging has been shown to play an important role in the primary staging of many cancers and has had a major impact on radiotherapy target volume definition [1]. On the other hand, while FDG PET/CT is reliable for remote nodal and distant metastases, it is less reliable for regional lymph node status [2, 3]. One key factor hindering interpretation of FDG-PET/CT is false-positive tracer uptake in acute inflammation. Recently, new targeting molecules based on a fibroblast activation protein specific enzyme inhibitor (FAPI) have been developed for diagnostic and therapeutic use as a tumor-specific tracer [4–6]. A recent clinical analysis of patients with lower gastrointestinal tract malignancies demonstrated that ^68^Ga-FAPI-PET/CT imaging led to changes in TNM classification relative to conventional imaging [7]. The aim of the current analysis was to evaluate the diagnostic impact of FAPI-PET/CT imaging for primary staging of esophageal cancer patients and its implications for target volume delineation of radiation therapy.

## Materials and Methods

### Patient Cohort

We retrospectively analyzed a cohort of seven patients with a new diagnosis of esophageal cancer. All patients were referred for molecular imaging by their treating radiation oncologists. The major intent of the study was to clarify possible regional lymph node involvement and tumor extent in the esophagus in order to improve target delineation. All patients gave written informed consent to undergo ^68^Ga-FAPI PET/CT on an individual-patient basis following the regulations of the German Pharmaceuticals Act §13(2b) (approval of the local ethics committee S016/2018). FDG-PET/CT imaging was not part of the routine staging.

### PET/CT Imaging and Image Evaluation

Linder *et al.* and Loktev *et al.* have previously described synthesis and labeling of ^68^Ga-FAPI-04 and ^68^Ga-FAPI-46 [8, 9]. All imaging data were acquired using a Biograph mCT Flow scanner (Siemens) according to scan protocols as previously published [10, 11]. After non-contrast-enhanced low-dose CT, PET scans were conducted in 3-dimensional mode (matrix, 200 × 200) followed by correction of emission data and reconstruction. ^68^Ga-FAPI-04 (with a specific activity of 20.6–37.2 GBq/mg) was used in 6 patients, and ^68^Ga-FAPI-46 (with a specific activity of 20.5–37.0 GBq/mg) in 1 patient with injected activities ranging from 180 to 325 MBq. PET acquisition was started 1 h after injection. We evaluated images and tracer uptake as previously published [7]. Volumetric quantification of macroscopic primary tumors (GTV, gross tumor volume) was performed with and without consideration of FAPI PET/CT imaging for all patients.

### Statistical Analysis

To analyze standard uptake values, median, standard deviation, and range were used. Mann-Whitney *U* tests were applied for tumor-to-background ratios (SigmaPlot 12.0), and Microsoft Excel for Mac version 16.35 was used for all other statistical analyses. GTVs were measured using Accuray Precision software.

## Results

Median age of the cohort was 63.5 years (range 57.8–82.8 years). Histologic diagnosis was squamous cell carcinoma (SCC) in six patients and adenocarcinoma (AC) in one patient. Six patients were referred to our radiation oncology department for definitive radiation in combination with chemotherapy, and one patient had an indication for neoadjuvant chemoradiation. All FAPI-PET/CT scans were conducted for primary staging and therapy planning. Patient characteristics are provided in Table 1***.***

**Table 1.** Patient characteristics

*n* = 7 esophageal cancer patients	
Age (years)	
Median	63.5
Range	57.8–82.8
Sex	
Male	5
Female	2
Histology	
SCC	6
AC	1
T stage	
Tx	1
T2	1
T3	3
T4	2
N stage	
N0	4
N1	2
N2	1
Treatment concept	
Neoadjuvant therapy	1
Definitive therapy	6
Gross tumor volume (GTV)	(cm^3^; median (range))
Standard CT scan	34.25 (13.27–106.9)
Considering FAPI-PET	37.73 (13.82–106.9)

### FAPI Uptake

All seven primary tumors in the esophagus were detected by FAPI PET/CT. In one patient, two bilateral supraclavicular lymph nodes and one lymph node in the mediastinum were FAPI positive but had not been identified on a previous CT scan. In another patient, regional lymph node metastases suspected on CT were confirmed by FAPI PET/CT. Another patient demonstrated suspicious tracer uptake in the left submandibular salivary gland (SUVmax 9.05; SUVmean 4.05). Following salivary gland scintigraphy and ENT-consultation, the presumed diagnosis was Sjogren’s disease without any sign of malignancy or metastasis. No lesions suspicious for distant metastases were detected in any patient.

Median SUVmax and SUVmean values for primary tumors 1 h after injection were 17.2 (range 5.7–23.3) and 8.6 (range 2.8–12.9), respectively. Lymph node metastases had median SUVmax and SUVmean values of 9.7 (range 6.0–13.4) and 5.1 (range 2.8–7.3). Regarding background activity, the average normal organ uptake was very low (myocardium, 1.7 [SUVmax] and 0.9 [SUVmean]; blood pool, 1.6 [SUVmax] and 1.2 [SUVmean]; normal liver parenchyma, 1.5 [SUVmax] and 0.8 [SUVmean]). Thus, excellent tumor-to-background ratios of more than 11 (tumor-to-blood pool, SUVmax, and SUVmean) could be achieved making nodal disease readily visible (Table 2).

**Table 2.** Standard uptake values (average)

Site	SUVmax	SUVmean
Primary tumor	16.48	8.61
Lymph node metastases	9.73	5.06
Brain	0.16	0.05
Oral mucosa	1.98	1.26
Parotis	1.82	1.16
Thyroid	2.64	1.39
Lung	0.56	0.37
Myocardium	1.67	0.90
Blood pool	1.60	1.17
Liver	1.51	0.83
Pancreas	2.32	1.47
Spleen	1.56	1.03
Kidney	1.97	1.19
Intestine	0.88	0.48
Muscle	1.52	1.04
Fat	0.63	0.36
Spinal canal	0.67	0.50
Bone	1.10	0.57

### Consequences for Radiotherapy Planning

**Fig. 1.**
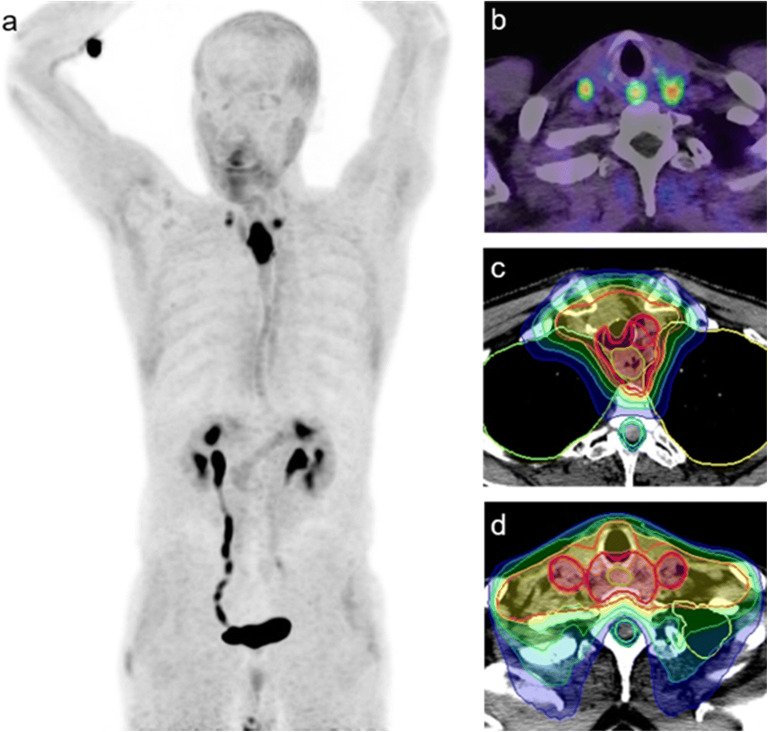
FAPI-guide radiotherapy with simultaneous integrated boots (**c**, **d**) to FAPI-positive lymph node metastases (**a**, **b**).

**Fig. 2.**
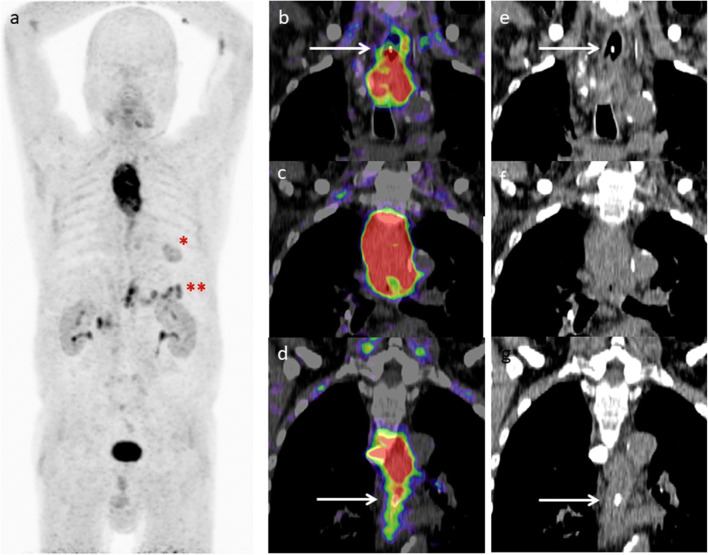
Patient with clipping of proximal and distal tumor margins (**e**–**g**) and correlating FAPI uptake (**b**–**d**). MIP (**a**) demonstrated non-malignant FAPI uptake within a cardiac scar after heart attack (*) and within pancreas to chronic pancreatitis (**).

All 7 patients had standard CT imaging for primary staging before they were referred for FAPI PET/CT. Three patients had endoscopic fiducial markers placed at the macroscopic tumor margins before start of therapy. Following simultaneous integrated boost (SIB) therapy for macroscopic tumor, FAPI PET/CT imaging led to dose escalation for lymph node metastases in 2 patients (Fig. 1) and eliminated boosts in 3 patients with multiple small lymph nodes in the mediastinum as these did not show any FAPI tracer uptake. Due to the very good tumor-to-background contrast ratio, FAPI PET/CT imaging improved target volume delineation in 6 out of 7 patients. In four patients, inclusion of FAPI PET/CT data into radiation therapy planning resulted in larger GTVs compared with planning with standard CT imaging, while for one patient, the volume was reduced (one patient without any changes). The median GTV based on FAPI PET/CT imaging was 37.73 cm^3^ (range 13.82–106.9) whereas it was 34.25 cm^3^ (range 13.27–106.9) without PET data (+ 10.2 %; Table 1). In 3 patients who underwent pretherapeutic endoscopic fiducial marking of tumor margins, conformity of tracer uptake and clipping distance was very well matched (Fig. 2).

## Discussion

Survival in esophageal cancer patients remains relatively poor, mostly due to high rates of local recurrence and distant metastases. For all stages, the 5-year overall survival is about 19 % [12]. There is some data indicating that dose escalated radiotherapy could improve outcomes [13]. On the other hand, reducing treatment-associated toxicity is a major goal of radiation treatment planning. To meet these competing demands, accurate delineation of tumor extent is essential.

To our knowledge, this is the first study evaluating the use of FAPI PET/CT in the primary staging of esophageal cancer patients for radiotherapy. Our first clinical experience with FAPI PET/CT staging demonstrated that ^68^Ga-FAPI were able to detect both primary tumors and lymph node metastases from esophageal cancer. The SUV values of the detected primary esophageal tumors were among the highest seen by Kratochwil *et al.* using FAPI PET/CT in a variety of tumor types [11]. Due to the very good tumor-to-background ratio, FAPI PET/CT has the potential to improve radiation therapy planning and thereby improve outcomes while reducing therapy-associated toxicity by adapting boost volume definition in preparation for external-beam radiotherapy.

Compared with FDG-PET/CT, the clinical assessment of quinoline-based FAPI tracers has shown several advantages such as fast renal clearance, equal or even better tumor-to-background contrast ratios, independence from blood glucose levels, and feasibility for quick image acquisition [10]. Recently, Chen *et al.* showed that ^68^Ga-FAPI-04 PET/CT had a higher detection rate of primary tumors and better sensitivity in the detection of lymph node, bone, and visceral metastases compared with ^18^F-FDG PET/CT in different types of cancer [14]. One of the major limitations of the FDG tracer remains its false-positive uptake in inflammation caused by an increased expression of glucose transporters in activated inflammatory cells [15]. Thus, differentiating nonmalignant from malignant tissue can be challenging. In contrast to FDG-PET/CT, FAPI-PET/CT is highly specific for tumors and tissues undergoing remodeling. In contrast, acute inflammatory disease does not result in high tracer uptake. However, FAPI can be taken up in chronic inflammatory conditions such as Sjogren’s syndrome seen in this case series [16, 17].

Despite the small cohort size, FAPI PET/CT staging resulted in alteration of radiotherapy planning in nearly all patients. Although the cohort is small, we were able to demonstrate promising visualization of primary tumors and lymph node metastases in esophageal cancer patients. Larger prospective studies will have to analyze the potential of FAPI PET/CT imaging to improve radiation therapy outcomes in esophageal cancer patients.

## Conclusion

Based on this small case series, the use of FAPI PET/CT for primary staging of esophageal cancer patients is promising and may be of particular benefit to radiation therapy planning. This early experience with this novel agent in esophageal cancer suggests high tumor uptake and low background activity, thereby facilitating tumor volume delineation in these patients. As with other tumor types, FAPI PET/CT could play an important role in improvement and personalization of oncologic treatment plans.
